# Lipid Biology of Archaea

**DOI:** 10.1155/2012/710836

**Published:** 2012-12-17

**Authors:** Angela Corcelli, Parkson Lee-Gau Chong, Yosuke Koga

**Affiliations:** ^1^Department of Basic Medical Sciences, University of Bari Aldo Moro, Piazza G. Cesare, 70124 Bari, Italy; ^2^Department of Biochemistry, Temple University School of Medicine, 3420 North Broad Street, Philadelphia, PA 19140, USA; ^3^Department of Chemistry, University of Occupational and Environmental Health, Yahatanishi-ku, Kitakyushu 807-8555, Japan

Today, there is increasing awareness of the multiple dynamic roles of lipids in cell life. 

Knowing how lipid molecular species are organized, interact with proteins, and change with environmental stress and metabolic state is crucial to understanding the membrane structure and the cellular functions.

The present view of lipid biology arises from the availability of technologies able to detect even minor lipid components with short lifetimes. In the late 1990s, the technical innovation in mass spectrometry led to the development of “lipidomics” as an evolution of lipid biochemistry. Furthermore, progress in microscopy and the availability of many types of fluorescent probes, together with genetic engineering, presently offer the possibility to move quickly towards lipid systems biology. As a consequence, besides being able to analyze major and minor lipids of all structures and sizes by mass spectrometry and nuclear magnetic resonance, we can study topology, structural organization, and dynamics of lipids by microscopy and fluorescent probes in living cells and microbes. 

Lipids are among the taxonomic traits that can be used to clearly delineate the archaea from all other organisms. Archaeal phospholipids are built on glycero-1-phosphate and contain ether-linked isoprenoid chains, while bacterial and eukaryal lipids are constituted of fatty acids ester-linked to glycero-3-phosphate. The radical structural differences between lipids of archaea and bacteria or eukaryotes raise many questions about early evolution of cell membranes.

This special issue contains selected papers dealing with archaeal phospholipid biosynthetic pathways, physical chemical properties and biotechnological applications of archaeal lipids, mass spectrometry lipid analyses and lipids of the archaeal viruses.

An updated phylogenic analysis of enzymes involved in archaeal phospholipid biosynthetic pathways is presented by the group of D. Moreira. The biosynthetic pathways have also been analyzed from the experimental point of view in a study of the group of H. Hemmii, which has expressed 4 genes involved in the biosynthesis of archaeal phospholipids in *E. coli* resulting in the production of archaeal-type lipids in the bacterium; in the future such engineered *E. coli* cells may serve to test the properties of mixed membranes constituted of both archaeal and bacterial phospholipids.

Lipid components of the membranes of *Pyrococcus furiosus* have been analyzed by MALDI/TOF-MS coupled to TLC in a study of S. Lobasso et al.; while lipids of uncultured methanogens present in geological samples have been detailed characterized by ESI-MS by M. Y. Yoshinaga et al.

The ability of archaeal lipids to act as adjuvant in vaccines designed to give protection against solid tumor has been examined in a study by G. D. Sprott et al.

A review article by E. Roine and D. H. Bamford describes the specific lipid components of viral membranes of archaeal viruses, in the frame of their possible functional roles in the mechanism of infection.

The chemical physical properties of the lipid core of membranes constituted of archaeal diether lipids have been examined in a study by A. Ota et al. by using fluorescence steady-state anisotropy and electron paramagnetic resonance measurements and in a review article of P. L. Chong et al. where the role of cyclopentane rings in the lipid chains of tetraether lipids on membrane fluidity is examined.

Finally an interesting contribution of Y. Koga comparatively examines the different mode of adaptation to high temperature of archaea and bacteria pointing out the role that differences in chemical physical properties of archaeal and bacterial lipids might have played in the differentiation of archaea and bacteria during evolution.

We hope that this issue will contribute to the understanding of the multiple roles of lipids in the cell biology of archaea and will stimulate further investigation in this field, opened by the pioneering studies of Morris Kates in the 60s.

*Angela Corcelli* *Parkson Lee-Gau Chong* *Yosuke Koga*

